# Impact of semaglutide withdrawal on cardiometabolic profile and physiology of energy balance: Recovery effects after semaglutide termination – The REST trial study protocol

**DOI:** 10.1371/journal.pone.0354237

**Published:** 2026-07-27

**Authors:** Taras Yevusiak, Alanna Weisman, Ravi Retnakaran, Sean Wharton, Daniel J. Drucker, Caroline K. Kramer

**Affiliations:** 1 Leadership Sinai Centre for Diabetes, Mount Sinai Hospital, Toronto, Canada; 2 Lunenfeld-Tanenbaum Research Institute, Mount Sinai Hospital, Toronto, Canada; 3 Division of Endocrinology, University of Toronto, Toronto, Canada; 4 Wharton Medical Clinic Weight and Diabetes Management, Burlington, Ontario, Canada; PLOS: Public Library of Science, UNITED STATES OF AMERICA

## Abstract

**Background:**

Glucagon-like peptide-1 (GLP-1) receptor agonists such as semaglutide have been shown to induce substantial weight loss and improve cardiometabolic risk factors in patients living with obesity. However, most individuals regain weight after abrupt withdrawal of semaglutide, with reversal of its beneficial cardiometabolic effects.

**Methods:**

We propose a randomized controlled trial to determine whether a gradual dose reduction of semaglutide prior to complete discontinuation is associated with differential changes in weight and cardiometabolic profile as compared to immediate treatment cessation. Individuals living with obesity without preexisting cardiovascular disease who are receiving semaglutide for weight management and have achieved prior weight reduction of at least 10% with no further weight loss over past 12 weeks will be randomized to either gradual dose reduction over 16 weeks or abrupt treatment discontinuation. The primary outcome will be the difference in body weight change (%) between the study groups. Secondary outcomes will include 24-hour ambulatory blood pressure and fasting ghrelin levels.

**Discussion:**

We hypothesize that gradual reduction of semaglutide will be associated with less weight regain and cardiometabolic deterioration compared with immediate cessation. This study addresses an important real-world problem regarding treatment discontinuation strategies and may inform future approaches for long-term obesity management.

**Trial registration:**

NCT07294950 (ClinicalTrials.gov).

## Introduction

Obesity, defined by body mass index (BMI) ≥30 kg/m^2^, is a complex chronic disease that has become a major public health problem. According to Canadian statistics, approximately 63.1% of adults are living with overweight or obesity, which has been linked with multiple comorbidities [1–3]. Importantly, higher BMI has been associated with poorer cardiovascular risk profiles and increased mortality regardless of the presence of obesity-related metabolic complications such as diabetes and hypertension [4,5].

Lifestyle modification consisting of medical nutrition therapy and physical activity combined with adjunctive pharmacotherapy has been recommended in the clinical management of obesity [6]. Although modest weight reductions of 3–5% of initial body weight can result in significant improvement in obesity-related comorbidities, adjunctive pharmacotherapy has been recommended to achieve a greater degree of weight reduction and prevent weight regain [6–8]. In this context, semaglutide, a glucagon-like peptide-1 receptor agonist, has been shown to induce substantial weight loss and improve cardiometabolic parameters. In clinical trials, semaglutide promoted mean weight loss of approximately −16.9% and had beneficial effects on blood pressure, glucose metabolism, inflammatory markers, and lipid profiles [9–13]. However, most individuals regain weight after abrupt withdrawal of semaglutide, with approximately two-thirds of the weight lost being regained within one year [11–12]. This rebound effect is likely related to physiologic mechanisms triggered by weight reduction that induce compensatory changes in energy balance homeostasis, including increased appetite and reduced energy expenditure [14–17].

There is little clinical data on the best strategy to achieve weight maintenance and cardiometabolic benefits following semaglutide discontinuation. This raises the question of whether a gradual de-escalation of semaglutide could ameliorate the tendency for weight regain and cardiometabolic deterioration. Thus, we propose a randomized controlled trial to determine whether a gradual dose reduction of semaglutide prior to complete discontinuation is associated with differential changes in weight and cardiometabolic profile as compared to immediate treatment cessation.

## Materials and methods

### Study design

**Fig 1:**
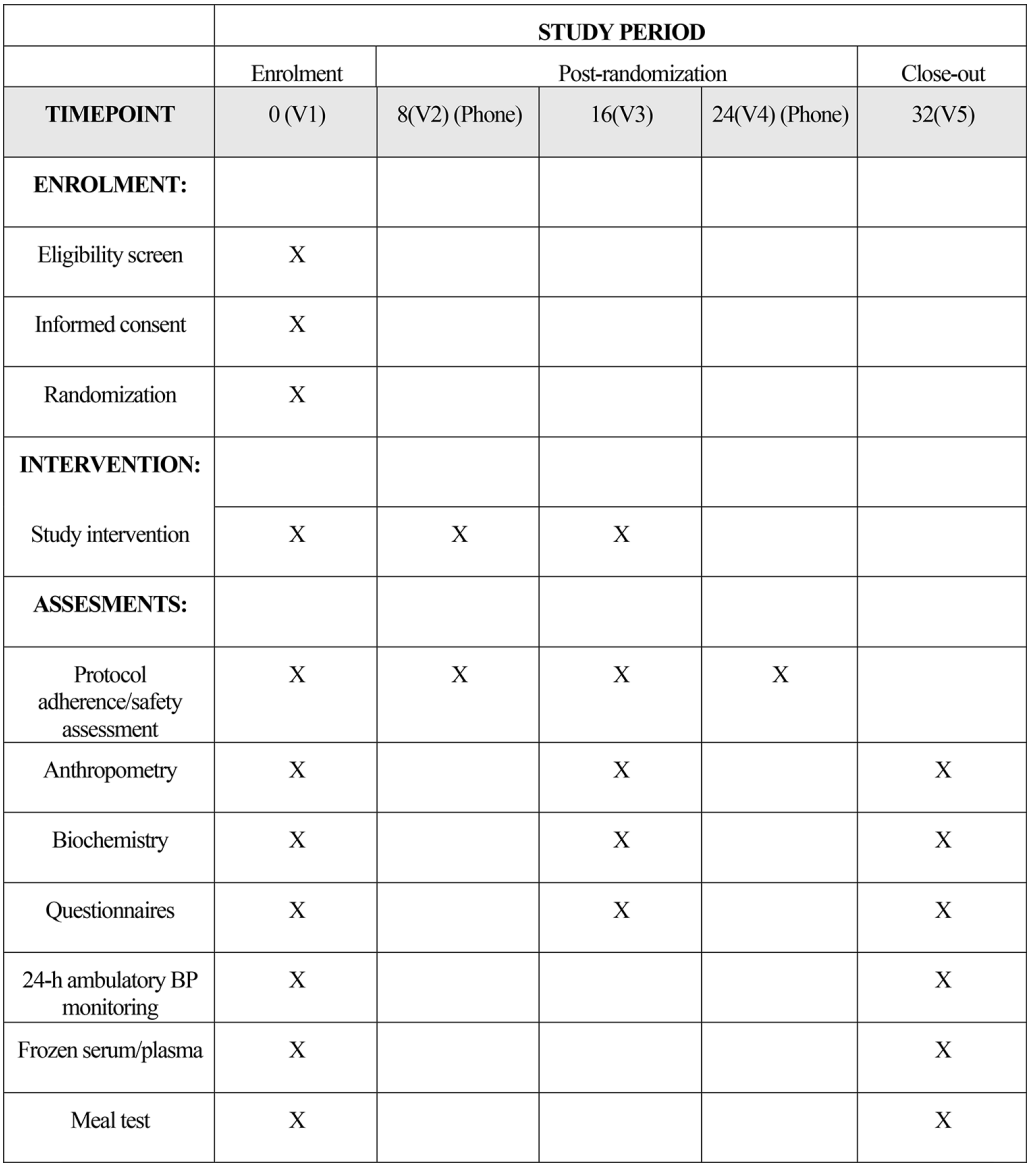
Participant timeline: Schedule of enrollment, interventions, and assessments.

**Fig 2:**
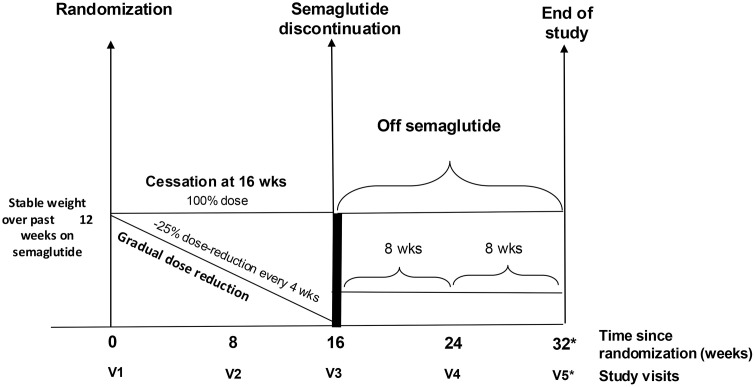
Schematic of study design: randomized controlled trial to evaluate the impact of semaglutide withdrawal (gradual dose reduction versus immediate treatment cessation) on weight changes and cardiometabolic outcomes.

We propose an open-label, parallel-arm, randomized controlled trial designed to evaluate the impact of semaglutide discontinuation strategies on weight and cardiometabolic outcomes. The total study duration will be 32 weeks, including a 16-week treatment discontinuation phase followed by a post-discontinuation follow-up period (Figs 1 and 2).

### Participants

Men and women aged 18–75 years with obesity, or overweight with adiposity-related complications, will be eligible for inclusion. Participants will be required to be receiving weekly subcutaneous semaglutide at a minimum dose of 1 mg, to have achieved at least 10% weight reduction from pre-treatment body weight, and to have demonstrated stable weight over the preceding 12 weeks. Individuals with preexisting cardiovascular disease, type 2 diabetes, pregnancy, prior bariatric surgery, eating disorders, or significant renal, hepatic, or malignant disease will not be considered eligible (Table 1).

**Table 1:** Inclusion and exclusion criteria.

***Inclusion Criteria***
• Men and women with previously diagnosed BMI ≥ 30 kg/m^2^ or BMI ≥ 27 kg/m^2^ and adiposity-related complications (such as osteoarthritis, nonalcoholic liver disease, sleep apnea, and hypertension) without preexisting cardiovascular disease or type 2 diabetes.
• Age 18–75 years inclusive • Ongoing weight-loss treatment consisting of weekly subcutaneous semaglutide at minimum dose of 1 mg/weekly with documented weight reduction of at least 10% of pre-treatment body weight • Stable weight over past 12 weeks (less than 5% change in body weight) (self-reported) • Ability to read and understand English
***Exclusion Criteria***
• Previously diagnosed cardiovascular disease defined as previous myocardial infarction, previous stroke, or symptomatic peripheral arterial disease. • Currently pregnant or lactating • Previously diagnosed type 2 diabetes • Use of any other pharmacological treatment for weight-loss • Previous surgical treatment for weight loss such as gastric bypass or gastric band
• Any history of eating disorder
• Renal dysfunction as evidenced by estimated glomerular filtration rate < 25 ml/min by CKD-EPI Creatinine Equation • New York Heart Association class II-IV heart failure
• Hepatic disease considered to be clinically significant (includes jaundice, chronic hepatitis, or previous liver transplant) or transaminases > 2.5X the upper limit of normal
• Malignant neoplasm requiring chemotherapy, surgery, radiation or palliative therapy within the previous 5 years (with the exception of basal cell skin cancer) • Personal or family history of medullary thyroid carcinoma or in patients with Multiple Endocrine Neoplasia syndrome type 2 • Any other factor likely to limit adherence to the study, in the opinion of the investigators

### Randomization and interventions

Participants will be randomized in a 1:1 ratio using a random number generator to one of two semaglutide discontinuation strategies: (i) immediate cessation, in which participants will continue their current dose for 16 weeks followed by complete discontinuation, or (ii) gradual dose reduction, in which the semaglutide dosage will be reduced by 25% every 4 weeks until complete discontinuation at week 16. All participants will receive standard lifestyle recommendations, including physical activity, portion control, and self-monitoring of weight.

### Follow-up and assessments

Participants will undergo study visits at baseline, 16 weeks, and 32 weeks, with interim phone assessments. Measurements will include anthropometry, fasting biochemical analyses, questionnaires assessing eating behavior, and 24-hour ambulatory blood pressure monitoring. Specifically, biochemistry will include fasting glucose, hemoglobin A1c, lipid profile, C-reactive protein, complete blood count, electrolytes, creatinine and liver enzymes. Control of Eating and appetite questionnaires will be completed to evaluate eating pattern. Nutritional intake will be assessed by a validated automated 24-hour self-administered dietary tool to be completed on-line during the in-person study visits (https://asa24.nih.gov/demo/) [18]. Once the participant completed the food record the data which is de-identified, the information will be downloaded by the research coordinator and kept at Sinai server. 24-h ambulatory BP monitoring (ABPM) will be obtained by oscillometry (Spacelabs Healthcare device with calibration certification), with a 30- min interval in the daytime and 60-min interval in the nighttime period. ABPM will be performed on an ordinary workday, and participants will be advised to maintain their usual daily activities. This monitoring enables the recording of the means of 24-h, daytime, and nighttime systolic and diastolic BP as well as systolic and diastolic BP loads, pulse pressure, and the overnight dipping pattern. It also enables the diagnosis of hypertension, white coat hypertension, and masked hypertension. A standardized meal test will enable assessment of the post-prandial response of hormones involved in appetite regulation (including ghrelin that is a secondary outcome). After a minimum of 8-hour overnight fast, participants will be provided with a standard breakfast (commercially available shake) containing ~500–600 kcal, with approximately 51% of the energy from carbohydrate, 33% from fat, and 16% from protein. Blood samples will be collected at fasting and at 30, 60, 90, 120, and 150 minutes after the meal. Frozen samples will be obtained for the future assessment of satiety hormones, emerging cardio-metabolic risk factors, biomarkers, proteomics and metabolomics relevant to the current research question. These samples will be de-identified and kept at a -80C freezer at Lunenfeld Taunenbaum Research Institute under the Principal Investigator laboratory. In case of a later consent withdrawal, samples will be disposed according to the standards of our institution. Visual analogue scales will be used to evaluate appetite and desire to eat at each timepoint [19].

### Outcomes

The primary outcome will be the difference in percentage change in body weight between baseline (treatment discontinuation) and 16 weeks after complete semaglutide withdrawal. Secondary outcomes will include differences in 24-hour systolic blood pressure and fasting ghrelin levels at 16 weeks after discontinuation. Additional outcomes will include other anthropometric measures, energy balance hormones, and blood pressure regulation parameters (Table 2).

**Table 2:** Primary and secondary outcomes.

*Outcome*	*Description of measure*
**Primary**	
Differences in changes in body weight between each study group	Differences in body weight change (%) between baseline visit 1 (semaglutide discontinuation) and 5 (16-weeks after complete semaglutide withdrawal) will be compared between the study groups
**Secondary**	
Differences in 24-h systolic BP levels between each study group	Systolic BP will be assessed as the difference in 24-h ambulatory systolic BP between the study groups at visit 5 (16-weeks after complete semaglutide withdrawal)
Differences in fasting ghrelin between each study group	Differences in fasting ghrelin between the study groups will be assessed at week 5 (16-weeks after complete semaglutide withdrawal)
**Ancillary**	
Additional anthropometric measures	Changes in BMI [calculated as weight / height^2^ (in kg/m^2^)] and waist circumference between baseline visit 1 (semaglutide discontinuation) and 5 (16-weeks after complete semaglutide withdrawal) will be compared between the study groups; changes in body weight change (%) between baseline visit 1 (semaglutide discontinuation) and 5 (16-weeks after complete semaglutide withdrawal) will be compared between the study groups to account for changes in weight that occurred during treatment discontinuation period (first 16-weeks).
Additional measures of energy balance regulation	Changes in ghrelin, peptide YY, GLP-1, glucose-dependent insulinotropic polypeptide, and glucagon responses to a meal test [calculated as incremental area-under-curve by trapezoidal rule] between study visit 1 (semaglutide discontinuation) and 5 (16-weeks after complete semaglutide withdrawal) will be compared between the study groups.
Additional measures of BP regulation	Differences between the study groups in 24-h diastolic BP, daytime and nighttime systolic and diastolic BP levels as well as the prevalence of hypertension, white coat hypertension, and masked hypertension at week 5 (16-weeks after complete semaglutide withdrawal) will be compared between the study arms.

### Sample size and statistical analysis

A total sample size of 98 participants is estimated to provide 80% power to detect a 3% difference in weight change between groups, accounting for a 10% loss to follow-up. Comparisons between groups will be performed using Student’s t test. Longitudinal changes in body weight will be evaluated using generalized estimating equation models to assess treatment effects, time effects, and treatment-by-time interactions. Statistical analyses will be conducted after completion of all study visits, with no interim analysis planned.

### Setting and recruitment

This trial will take place at a single academic center, Mount Sinai Hospital (MSH) in Toronto, Canada. It has received institutional research ethics and board approval (#REB 1360) and all participants will provide informed consent form. We will conduct recruitment by contacting eligible participants at MSH, recruiting through nearby community physicians’ offices, and advertising with social media. Initial contact with interested participants will be made by one of the patient’s healthcare providers. Following the first interaction, the individuals will then be contacted by our research coordinator to determine eligibility. Potential participants will be emailed a consent form and provided with as much time as needed to review and decide on whether or not they wish to participate. This study has just started recruitment on 01/March/2026 and has estimated completion date of recruitment and follow up (data collection) of 31/July/2028 with completion of data analysis and reporting of results expected for 2030.

### Knowledge dissemination

We will work with patient partners to ensure that the research results are communicated in a manner that is easily accessible to the patients’ community. For researchers, we plan to make presentations at the American Diabetes Association (ADA) and Diabetes Canada annual meetings as well as publish scientific papers. Patients will be informed about our findings at the end of the study through a newsletter sent out to all participants. Lastly, the general public will be informed of our findings through forms of media such as newspaper coverage, online articles, and television outlets.

No datasets were generated or analyzed during the current study protocol. Data will be made available upon study completion.

### Future perspective

This randomized controlled trial is designed to evaluate whether a gradual dose reduction of semaglutide prior to discontinuation is associated with improved weight maintenance and cardiometabolic outcomes compared to immediate cessation. Based on existing evidence, it is anticipated that participants undergoing immediate treatment cessation will experience significant weight regain, consistent with previous trials demonstrating that a substantial proportion of lost weight is regained following withdrawal of pharmacological therapy [11,12]. In contrast, gradual dose reduction may attenuate this effect by allowing physiological adaptation in energy balance regulation.

It is expected that differences in body weight change between the study groups will emerge within 12–16 weeks following treatment discontinuation, a period during which the steepest rebound in weight has previously been observed. In addition to weight outcomes, gradual dose reduction is hypothesized to result in more favorable cardiometabolic profiles, including lower 24-hour systolic blood pressure and improved regulation of appetite-related hormones such as ghrelin. These effects may reflect a reduced compensatory response in appetite stimulation and energy conservation that typically follows rapid weight loss or abrupt cessation of therapy [17,20–22].

The findings of this study will address an important gap in the literature, as previous trials have primarily focused on continued treatment versus abrupt discontinuation, without evaluating alternative discontinuation strategies. Importantly, the high real-world rates of treatment discontinuation highlight the clinical relevance of identifying strategies that preserve therapeutic benefits after stopping medication [22]. This study has important implications for clinical practice and future research. If gradual dose reduction is shown to mitigate weight regain and cardiometabolic deterioration, it may represent a pragmatic and lower-cost approach to long-term obesity management. This would be particularly relevant for individuals facing financial barriers or limited access to long-term pharmacotherapy. Additionally, these findings could inform the design of larger trials aimed at optimizing treatment duration and discontinuation strategies for incretin-based therapies.

## Conclusions

Our proposed trial evaluates a novel approach to semaglutide discontinuation in individuals living with obesity without preexisting cardiovascular disease. By comparing gradual dose reduction with immediate cessation, it aims to determine whether a de-escalation strategy can improve weight maintenance and cardiometabolic health following treatment withdrawal. The results are expected to provide important insights into the physiology of weight regain and inform more sustainable and accessible approaches to obesity management.

## Supporting information

S1 Table SPIRIT checklist. (DOCX)
